# Obesity-Related Metabolic Syndrome: Mechanisms of Sympathetic Overactivity

**DOI:** 10.1155/2013/865965

**Published:** 2013-10-31

**Authors:** Maria Paola Canale, Simone Manca di Villahermosa, Giuliana Martino, Valentina Rovella, Annalisa Noce, Antonino De Lorenzo, Nicola Di Daniele

**Affiliations:** ^1^Division of Hypertension and Nephrology, Department of System Medicine, University of Rome Tor Vergata, Rome, Italy; ^2^Division of Clinical Nutrition and Nutrigenomic, Department of Biomedicine and Prevention, University of Rome Tor Vergata, Rome, Italy

## Abstract

The prevalence of the metabolic syndrome has increased worldwide over the past few years. Sympathetic nervous system overactivity is a key mechanism leading to hypertension in patients with the metabolic syndrome. Sympathetic activation can be triggered by reflex mechanisms as arterial baroreceptor impairment, by metabolic factors as insulin resistance, and by dysregulated adipokine production and secretion from visceral fat with a mainly permissive role of leptin and antagonist role of adiponectin. Chronic sympathetic nervous system overactivity contributes to a further decline of insulin sensitivity and creates a vicious circle that may contribute to the development of hypertension and of the metabolic syndrome and favor cardiovascular and kidney disease. Selective renal denervation is an emerging area of interest in the clinical management of obesity-related hypertension. This review focuses on current understanding of some mechanisms through which sympathetic overactivity may be interlaced to the metabolic syndrome, with particular regard to the role of insulin resistance and of some adipokines.

## 1. Introduction

The metabolic syndrome (MetS) is a cluster of abnormalities that include diabetes mellitus (DM), morbid obesity, dyslipidemia, and hypertension (HT) that are all risk factors for the development of cardiovascular disease (CVD) and chronic kidney disease (CKD) [1]. The National Cholesterol Education Program's Adult Treatment Panel III report (ATP III) identified six components of the MetS that relate to CVD: abdominal obesity, atherogenic dyslipidemia, raised blood pressure (BP), insulin resistance (I.R.)/glucose intolerance, and proinflammatory and prothrombotic state [2]. A major problem concerning the WHO and NCEP ATPIII definitions was their applicability to different ethnic groups, especially when obesity cutoffs were to be defined. This is particularly evident for the risk of type II DM, which may be associated with much lower levels of obesity in Asians compared to Caucasians. The International Diabetes Federation has then proposed a new set of criteria with ethnic/racial specific cutoffs [3]. The MetS central feature is obesity, and the MetS is a growing epidemic in the United States and throughout the world [4, 5]. Approximately 1 adult in 4 or 5, depending on the country, has the MetS. Incidence increases with age; it has been estimated that in people over 50 years of age, the MetS affects more than 40% of the population in the United States and nearly 30% in Europe [6, 7]. Whether the effects of the MetS are due to a sum of comorbidities or to individual features is still a matter of debate; however, there is sufficient data to support an increased risk of CVD in people affected by the MetS in the absence of other baseline risk factors [8–10]. Central obesity is an independent risk factor for CVD and is associated with MetS [11]. Central obesity predisposes to diabetic nephropathy, hypertensive nephrosclerosis, and focal segmental glomerulosclerosis and represents an independent risk factor for the development and progression of CKD [12]. Obesity and the development of I.R. are thought to be a central feature, contributing to the significant morbidity and mortality associated with the MetS and development of a particular resistant form of HT [13–15]. The development of resistant HT in individuals with MetS can be attributed to a number of factors including proinflammatory cytokines, inappropriate activation of the renin-angiotensin system (RAAS), vasoconstriction from increased sympathetic nervous system (SNS) activation, and dysregulation in adipokines production and secretion [16]. Several components of the MetS are associated with indirect or direct markers of adrenergic overdrive [17]. This review will focus on current understanding of the mechanisms through which sympathetic overactivity may be interlaced to the metabolic syndrome, with particular regard to the role of insulin resistance and of some adipokines.

## 2. Pathophysiology of the Mets

In 1988, Reaven first postulated “the syndrome X,” which is now named “Metabolic Syndrome” (MetS) [14]. Reaven noticed the frequent association of factors leading to the development of CVD: glucose intolerance, hyperinsulinemia, high serum triglycerides, low serum high-density lipoprotein cholesterol, and HT. I.R. was proposed as the “driving force” of the syndrome [14, 18]. Subsequently, other abnormalities, in particular prothrombotic and chronic proinflammatory states, were added to the definition of the MetS. Later on, abdominal obesity became the “core” of the syndrome [19–21]. Since metabolic abnormalities linked to I.R. are usually found in patients with abdominal obesity [22, 23] I.R. is considered to be the “core” of the MetS and central obesity its most important clinical clue [24].

## 3. Metabolic Syndrome and Sympathetic Overactivity

As BP and thermogenesis are both under adrenergic control, an alteration in the SNS could be part of the pathophysiology of the MetS. Also, alterations in the sympathetic control of heart rate (HR), cardiac output, peripheral vascular resistance, and renal sodium handling may promote, alone or in combination, the development and progression of HT [25, 26]. Actually, sympathetic overdrive occurs in MetS. Many components of the MetS are characterized by an increased adrenergic activity. Interestingly, sympathetic overdrive is detectable in obese patients prone to MetS before HT occurs. Also, when obesity and HT are both present in the same patient the degree of sympathetic activation is much greater than in those with either condition separately [27]. Individuals with central obesity show increased sympathetic nervous activity (SNA) when compared to individuals with subcutaneous form of obesity [28]. Increased sympathetic outflow has been reported in obese nonhypertensive individuals with the determination of circulating catecholamines, urinary norepinephrine (NE), muscle sympathetic nerve activity (MSNA) recordings of postganglionic sympathetic nerve fibers, and renal NE spillover [29], suggesting that vasoconstriction and renal mechanisms are both involved [30]. There is also evidence that SNS overactivity is not generalized in obesity [31]. Obesity causes differential activation of tissue SNS activity. Increase in HR results from a decrease in parasympathetic activity rather than an increase in sympathetic activity. On the contrary, SNA increases both in the kidney and skeletal muscles of obese hypertensive subjects. Increased SNA does not directly determine vasoconstriction but instead stimulates renin secretion and increases renal sodium reabsorption [15]. Interestingly, Kalil and Haynes showed that the presence of misleading inferences from multifiber recordings of MSNA involved vascular tone in obesity and also that increased SNA does not necessarily translate into increased vascular tone [30]. It is worth noting that MSNA was mainly measured rather than renal SNS activity, the most important pathway for SNS to cause chronic HT [15]. So MSNA activity may not reflect renal sympathetic nervous activity. Renal sympathetic nerves mediate most, if not all, of the chronic effects of SNA on BP in obesity. In obese dogs fed with a high-fat diet, bilateral renal denervation greatly attenuates sodium retention and HT. Thus, obesity may increase renal sodium reabsorption and cause HT mainly by increasing renal sympathetic activity [15].

## 4. Mechanisms of Sympathetic Overactivity in the Metabolic Syndrome

Among the different hypotheses which have been proposed to explain the cause of obesity and obesity-related metabolic disturbances, SNS activation plays thus a pivotal role. A large body of evidence clearly shows that sympathetic activity is increased in human obesity [32, 33]. In 1986, Landsberg suggested that sympathetic activation could represent an insulin-mediated adaptative response to overeating promoting thermogenesis and acting as a buffer against weight gain [34]. Reaven first proposed I.R. as the key abnormality leading to hyperinsulinemia, sympathetic activation, and HT [14]. Later on, other investigators stressed the important role of I.R. and hyperinsulinemia and their relationship to SNS activation [35, 36].

Interestingly, Julius et al. proposed increased sympathetic activity as the primary defect leading to I.R. and weight gain [35]. Whether SNS activation is the cause or the consequence of obesity is still matter of debate. SNS activation results in the release of norepinephrine which stimulates adrenergic receptors. Physiological responses depend upon the receptors present in the target organs, the fasting state, and the rate of neuronal firing [37]. Cardiovascular, renal, and metabolic effects of chronic and sustained SNS activation may contribute to HT and the development of I.R. over a prolonged period of time [37]. The pathophysiological mechanisms linking SNS overactivity and obesity-related MetS are complex and still need to be fully elucidated.

Multiple neurohumoral mechanisms can activate the SNS in patients with the MetS. Neural mechanisms include direct activation of the SNS in response to the activation of higher cerebral nuclei by hunger or feeding and renal afferent nerve activation mediated by perirenal fat accumulation and kidney compression [15]. Sympathetic activation can also be triggered by reflex mechanisms (arterial baroreceptor impairment), psychological stress, oxidative stress, obstructive sleep apnea, inflammation, and metabolic factors as I.R. and dysregulated production and secretion of adipokines from visceral fat with a particular important role of leptin.

**Figure 1:**
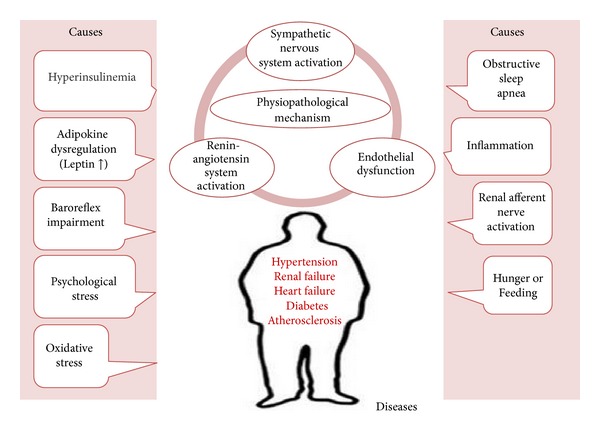
In obesity, infiltration of inflammatory cells in the white adipose tissue disturbs the secretion of adipokines and increases the activity of adipocyte renin-angiotensin system. Increased secretion of leptin and proinflammatory cytokines and decreased amounts of adiponectin contribute to the development of obesity-related hypertension.

Table 1 summarizes the possible causes and clinical effects of the MetS. We will first focus on the role of I.R./hyperinsulinemia, the key metabolic alteration in MetS, and then discuss the role of adipokines in the activation of SNS and their interplay with insulin.  Figure 1 summarizes these interactions.

## 5. Hyperinsulinemia and Sympathetic Overactivity

Hence, insulin plays a pivotal role in the development of DM, HT, and the MetS [38]. Insulin stimulates SNS to increase cardiac output and the delivery and enhances the utilization of glucose in the peripheral tissues [39]. I.R. is the inability of insulin to produce its numerous actions, in spite of its normal secretion from the pancreatic beta cells [15, 40]. Insulin elicits its various biological responses by binding to a specific receptor [41, 42]. The ability of insulin receptor to autophosphorylate and then phosphorylate intracellular substrates is crucial for the complex cellular responses to insulin [41–43]. The two major signaling pathways activated by insulin binding to its receptor, the phosphatidylinositol-3′-kinase (PI3K) pathway and the mitogenic-activated protein kinase (MAPK) pathway, among their effects, play a role in vasodilatation and in the decrease in nitric oxide (NO) production, respectively, [43, 44]. In the MetS I.R. mainly results from an impairment in the cellular events distal to the interaction insulin/insulin surface receptor [14]. Selective I.R., located primarily in the muscle and the adipose tissue, causes compensatory hyperinsulinemia which has an adverse impact on insulin-sensitive tissues [41, 42]. I.R. arises due to various genetic and acquired factors, including obesity [45]. The effects of insulin on BP are multifactorial, including sympathetic activation and direct antinatriuretic action [43]. In animal studies insulin increases sympathetic outflow via intracerebral administration [46, 47]. Recent investigations from Cassaglia and coworkers identify the arcuate nucleus, via the paraventricular nucleus of the hypothalamus, as the central site of action of insulin in the increase of SNS activity and in the sympathetic baroreflex gain [48]. Insulin receptors in the hypothalamus coactivate the SNS through a transport-mediated uptake of peripheral insulin across the blood-brain barrier [49]. Also, the presence of highly permeable capillaries in the arcuate nucleus (AN) allows insulin to activate receptors without a specific transport mechanism [50, 51]. In 1991, Anderson and coworkers showed that hyperinsulinemia causes sympathetic activation in humans [52]. Sympathetic overactivity occurring in the MetS is dependent on hyperinsulinemia and related I.R. [53]. Human studies suggest that hyperinsulinemia may contribute to the increased SNS activity observed in the obese MetS patients. Insulin secretion following a meal [54–56] or during a hyperinsulinemic euglycemic clamp [52, 57–60] determines an increase in MSNA and enhances the arterial baroreflex gain of SNA. Interestingly, chronic SNS overactivity contributes to a further decline of insulin sensitivity. Furthermore, SNS coactivation by the hypothalamic-pituitary-adrenal axis may also occur in the hyperinsulinemic state secondary to obesity [29]. In addition to the neural SNS overactivity at rest, obese subjects demonstrate baroreflex impairment and blunted responses to sympathoexcitatory manoeuvres. The exact cause of changes in baroreflex function in obese patients is not entirely clear. Changes in baroreceptor signalling may be a contributing factor to sympathetic overactivity as well as reduced baroreflex responsiveness [29]. Following hypocaloric diet, obese subjects showed a reduction in MSNA and whole body NE spillover rate [61]. However, during weight maintenance period following weight loss MSNA rebounded while NE spillover was preserved [29]. Recent findings from Straznicky et al. on obese MetS patients demonstrated that the progression to type II DM is associated with increased central sympathetic drive, blunted sympathetic responsiveness, and altered NE disposition [62]. Many studies showed that perturbed autonomic nervous system function in the MetS may be reversible. Recently, caloric restriction inhibited SNA via an antioxidant mechanism in the rostral ventrolateral medulla in obesity-induced hypertensive rats [63]. Also, data from Lambert et al. showed that in MetS patients dietary weight loss decreased sympathetic nerve firing and improved hemodynamic and metabolic parameters [64]. It is worth noting that in a very recent longitudinal investigation from Licht and coworkers, a dysregulation of autonomic nervous system in subjects under stress predicted 2-year development of the MetS [65]. However, other studies call into question the role of hyperinsulinemia in the determination of sympathetic overactivity. Obese subjects do not appear to retain their sensitivity to the stimulatory effects of insulin on SNS [29]. A greater increase in MSNA activity in response to euglycemic hyperinsulinemia was observed in lean subjects compared to obese by Vollenweider and coworkers [66]. Later on, similar results were obtained by Straznicky et al. in patients with I.R. and MetS, who exhibited a blunted sympathetic response to increased plasma insulin following a glucose load [67] consistent with central I.R. Certain adipokines expressed in central obesity may contribute to determining sympathetic overdrive and in case of overexpression may contribute to I.R., resulting in hyperinsulinemic state with greater sympathetic outflow [29].

Among the factors promoting the development of I.R. and progression of MetS, reactive oxygen species (ROS) play an important role. In obesity ROS are increased and ROS can be reduced with weight loss in humans [68]. In rats, obesity induced by a high-fat diet resulted in enhanced oxidative stress [69]. Ogihara and coworkers reported that ROS overproduction may cause insulin resistance in AngII-infused rats [70]. Blendea and coworkers confirmed these data and showed that insulin resistance was ameliorated by tempol in TG(mREN-2)27 (Ren-2) transgenic rats, which have a stimulated renin-angiotensin system [71]. Thus, ROS overproduction results in I.R. In mice, ROS overproduction in target organs of insulin, like adipose tissue and liver, preceded the onset of obesity and I.R. [72]. In a recent investigation, Yubero-Serrano and coworkers showed that the higher the number of MetS components, the greater the degree of oxidative stress, leading to increased plasma superoxide dismutase and glutathione peroxidase activities, plasma H_2_O_2_, lipid peroxidation products and sVCAM-1, and as well to decreased postischemic reactive hyperemia and total plasma nitrites. Also, oxidative stress increase was associated with higher body mass index, waist circumference, diastolic blood pressure, HOMAIR and triglycerides, glucose and insulin, and lower HDL-cholesterol and HOMAb and QUICKI indexes [73], confirming previous results from Fujita and coworkers [74].

## 6. Visceral Adiposity and MetS

Although adiposity is defined as an increase in total body mass, visceral fat expansion correlates with a cluster of metabolic abnormalities observed in the MetS [75]. Visceral fat represents a metabolically active organ and has been strongly related to insulin sensitivity [76, 77] and CVD both in humans and animals [77]. Subcutaneous fat, characterized by insulin-sensitive adipocytes, is mainly a fat depot. Instead, visceral fat adipocytes are insulin-resistant cells within a network of blood capillaries and infiltrating inflammatory cells [40]. Inflammatory cells within the visceral fat may play a role in adipocyte behaviour as a source of hormones and cytokines, called adipokines, with proinflammatory and proatherogenic action. Circulating cytokines including resistin and leptin are generally increased in obese subjects and in patients with DM [76, 78–80]. On the other hand, circulating adiponectin is decreased. Adiponectin is a tissue-specific circulating hormone with insulin-sensitizing and antiatherogenic properties. Also, adiponectin stimulates glucose and fatty acid oxidation in the muscle, enhances insulin sensitivity in the liver, increases free fatty acid oxidation, reduces hepatic glucose output, and inhibits monocyte adhesion and macrophage transformation to foam cells within the vascular wall [78–80].

I.R. and visceral obesity determine BP elevation by activating SNS and renin-angiotensin-aldosterone system (RAAS) [40], resulting in sodium retention and volume expansion as well as and endothelial and renal dysfunction [41, 44, 81]. Hyperinsulinemia activates RAAS in both heart and blood vessels, with production of angiotensin II which has proatherogenic effects. Angiotensin II inhibits vasodilator effects of insulin on blood vessels and glucose uptake into the skeletal muscle [41, 44, 82] resulting in decreased NO production, vasoconstriction, and GLUT 4 inhibition [40].

The presence of endothelial dysfunction in patients with obesity and I.R. was first reported by Steinberg and coworkers. Adipokines dysregulation and inflammatory state disrupt vascular homeostasis by causing an imbalance between the NO pathway and the endothelin 1 system, with impaired insulin-stimulated endothelium-dependent vasodilation [83].

It is noteworthy that in obesity vascular dysfunction also involves the other layers of the vessel wall. Obesity-induced changes in medial smooth muscle cells disrupt the physiological facilitatory action of insulin on the responsiveness to vasodilator stimuli, whereas the adventitia and perivascular fat appear to be a source of proinflammatory and vasoactive factors possibly contributing to the endothelial and smooth muscle cell dysfunction and to the pathogenesis of vascular disease as pointed out by Tesauro and coworkers [84, 85].

At the present time, body weight control has not yet proved to prevent metabolic and cardiovascular complications of obesity on a large scale. Recent data from the same investigators show that in the forearm circulation of hyperinsulinemic MetS patients, Rho-Kinase inhibition by fasudil improves both endothelium-dependent and independent vasodilator responsiveness, possibly by increased oxidative stress [86].

## 7. Effects of Adipokines on Sympathetic Overactivity

### 7.1. Adipokines

Adipose tissue produces bioactive substances, known as adipokines, and releases them into its direct surroundings and into the bloodstream [87]. Among the different actions of the adipokines is the regulation of arterial tone [80, 88]. Therefore, adipose tissue affects not only metabolism but also many functions of organs and tissues, such as brain, muscle, liver, and blood vessels. The presence of a normal amount of adipose tissue is essential. An imbalance can cause dysregulation in the release of adipokines that may result in vascular disturbances and inflammation [89]. Adipokines may alter SNS activity and impair insulin signaling. Adipokines mainly involved in obesity and MetS are leptin, nonesterified free fatty acids (NEFAs), reactive oxygen species (ROS), adipocytic angiotensinogen and resistin. A reduction of adiponectin may be involved as well. An unbalanced interplay between these adipokines may lead to an impaired insulin signaling as well as to a state of inflammation and/or alter sympathetic regulation [29]. The aim of this section is to focus on the main SNS-activating adipokine, leptin, and on the main SNS-inhibiting one, adiponectin.

### 7.2. Leptin and Leptin Resistance

Leptin is a 167 aminoacid-16 kDa protein. It is secreted from adipocytes proportionally to the adipose tissue mass [90–92]. Physiologically, leptin represents the inhibitory signal from fat that informs the brain about the body's stocks of stored energy [93]. Its ability to produce anorexigenic effects has been extensively studied and is beyond the scope of this review. Leptin decreases food intake, and this is particularly dependent on the depolarization and hyperpolarization of neurons in the AN of the hypothalamus [94, 95]. Leptin resistance develops in obesity because the ARH neurons expressing leptin receptors do not become further activated from baseline in response to exogenous leptin; consequently, increased leptin levels do not increase energy expenditure or decrease food intake [96, 97]. How leptin resistance develops and how it could be treated in obesity is now under investigation.

Whether SNA increase in obesity [31, 98, 99] leads to innervation of all organs or it is organ-specific is still matter of debate. An interesting finding, as it is now readily hypothesized, is that a chronic increase of SNA to the kidney contributes to the development of HT. HT is part of the MetS and is well known to contribute significantly to the development of CVD. Leptin acutely increases SNA [100, 101], although, at the present time, no conclusive data demonstrate that leptin increases SNA chronically, leading to HT. The increase of SNA observed in obesity also appears to cause organ damage, which exacerbates the risk of MetS and CVD [102]. When acutely injected into the dorsomedial hypothalamus of anesthetized rats leptin determines an increase in HR and BP [103]. Very recent animal studies show that leptin exerts its action at the level of the nucleus of the solitary tract where it alters the activity of neurons that mediate the cardiovascular responses to the activation of the aortic baroreceptor reflex [104] and in the forebrain to influence the baroreflex control of lumbar, renal, and splanchnic SNA and finally the HR [105]. Also, leptin activates brain centers that regulate SNS activity through a melanocortin-system-dependent pathway [106].

The interactions between the brain melanocortin system and leptin represent an important area of research to further understand the mechanisms leading to SNS activation in obesity.

Determining whether hyperleptinemia may be the cause of chronically elevated SNA in obesity, via activation of leptin receptors in higher brain regions, will hopefully lead to new treatment options for obesity. In a recent work, Curry and coworkers showed that a low MSNA and a lack of SNS-mediated support of resting energy expenditure 3 years after gastric by-pass should possibly be multifactorial in origin and involve changes in insulin sensitivity, body composition, and leptin [107].

Physiologically, leptin contributes to BP by its vasorelaxing and vasocontractile effects [108, 109]. While the contractile effect of leptin is attributed to SNS activation [110], various mechanisms seem to be responsible for leptin-induced vasorelaxation. This latter effect can be endothelium-dependent, either through the release of NO [110] or by other mechanisms [109, 111]. Recent findings from Schinzari and coworkers suggest that, under physiologic conditions, leptin stimulates both endothelin-1 and NO activity in the human circulation. This effect is absent in hyperleptinemic patients with MetS who are unresponsive to additional leptin [112].

### 7.3. Nonesterified Fatty Acids

Nonesterified fatty acids (NEFAs) act on all the aspects of glucose homeostasis, from uptake at peripheral tissue to hepatic production and disposal. NEFAs are increased in obesity and are inversely correlated with insulin sensitivity [113, 114]. Increased NEFAs may inhibit glucose uptake into peripheral tissues by impairing PI3-kinase activation [115]. PI3-kinase activation is lost indeed as a result of a high fat diet in mice [116]. Impaired PI3-kinase activation may also be due to excess of protein kinase C [117]. NEFAs can cross the blood-brain barrier, cause a central activation of MSNA in lean subjects [118, 119], and reduce cardiovagal baroreflex in lean and obese subjects [120]. NEFAs also stimulate plasminogen activator inhibitor-1, which may contribute to the association between increased plasma NEFA in obesity and the augmentation of MSNA [121]. However, other investigations demonstrated no significant effect of NEFAs on MSNA or sympathetic baroreflex sensitivity [122]. Furthermore, whole body and renal NE spillover did not change during infusion of NEFAs [123].

### 7.4. Adiponectin

Adiponectin is an anti-inflammatory, insulin-sensitizing, and antiatherogenic protein exclusively secreted by adipocytes. Adiponectin is an adipose tissue hormone, also known as gelatin-binding protein-28 (GBP28), AdipoQ, adipocyte complement-related protein (ACRP30), or apM1. Adiponectin circulates in trimeric, hexameric, and high-molecular-mass species. Different forms of adiponectin may play distinct roles in the balance of energy homoeostasis. Adiponectin is an insulin sensitizing hormone that exerts its action through its receptors Adipo R1, Adipo R2, and T-cadherin. Adipo R1 is expressed mainly in muscle, whereas Adipo R2 is predominantly expressed in the liver.

Adiponectin is inversely related to obesity, DM, and other I.R. states that cause metabolic dysfunction; adiponectin deficiency may also contribute to coronary heart disease, steatohepatitis, I.R., nonalcoholic fatty liver disease, and a wide array of cancers.

The human adiponectin gene was cloned through systematic sequencing of an adipose-tissue library [124, 125]. The apM1 gene encodes a 244 amino acid open reading frame containing a putative signal sequence repeat (66 amino acids) followed by a cluster of aromatic residues near the C terminus that shows a high local resemblance to collagens X and VIII and complement factor C1q [126].

The regulation of adiponectin receptors Adipo R1 and Adipo R2 is important to facilitate essential physiological functions. Adipo R1 is expressed ubiquitously and exhibits high affinity to the ligand, whereas Adipo R2 exhibits intermediate affinity. The expression of adiponectin and its receptors has been investigated in streptozotocin (STZ)-induced diabetic rat heart and in mouse skeletal muscle. STZ-induced DM upregulates adiponectin receptors in the heart [127].

Some evidence suggests that T-cadherin can bind to the hexameric and HMW forms of adiponectin but not to monomer globular and trimeric forms. T-cadherin is expressed ubiquitously, with the highest expression found in the heart and the aortic, carotid, iliac, and kidney arteries [128]. T-cadherin is bound to adiponectin and is critical for the association of adiponectin protection with cardiac stress in mice.

The pleiotropic roles of adiponectin have been studied in multiple *in vitro* and *in vivo* models. The multiple molecular targets of adiponectin mediate multiple pharmacological actions.

A steep rise in the prevalence of obesity has occurred over the past few decades. Obesity is inversely related to adiponectin, making adiponectin a negative marker of MetS. Furthermore, the expression of the receptors Adipo R1 and Adipo R2 declines by 30% in the subcutaneous fat of obese individuals, while they normalize following weight loss [129]. Adiponectin may play an important role in type II DM, HT, multiple sclerosis, and the dyslipidemias. The most significant role played by adiponectin is that of its insulin-sensitizing effect. Adiponectin in the diabetic's blood is lower than normal, whereas higher adiponectin in plasma minimizes the risk of type II DM [130]. Adiponectin relates negatively to blood glucose and insulin. Total adiponectin, HMW adiponectin, and the total/HMW ratio are all inversely related to homeostasis model assessment I.R. index. The total/HMW ratio is considered a better indicator of I.R. than total plasma adiponectin [131]. The role of adiponectin in I.R. was determined using knockout mice. These mice had normal plasma insulin, but its capability of lowering blood glucose was severely impaired, this clearly pointing to the role of adiponectin in glucose tolerance [132]. The absence of serum adiponectin in lipoatrophic mice causes hyperglycemia and hyperinsulinemia, which can be normalized by adiponectin injections. All studies on the putative role of adiponectin in IR and type II DM suggest that low plasma adiponectin causes susceptibility to these disorders.

In conclusion, adiponectin exerts an insulin-sensitizing action with profound effects on fatty acid oxidation and inflammation. Drugs affecting serum adiponectin may have a role in the treatment of type II DM and possibly the Mets. In obese adiponectin-knockout mice with HT adiponectin replenishment lowers elevated BP [133]. Existing drugs such as peroxisome proliferator-activated receptor agonists (thiazolidinediones), some angiotensin Adipo R1 receptor blockers (telmisartan), angiotensin-converting enzyme inhibitors, and cannabinoid Adipo R1 receptor blockers (rimonabant and taranabant) may increase circulating adiponectin [134]. However, future strategies should focus on upregulation of adiponectin/adiponectin receptors expression or on targeting adiponectin receptors with specific agonists [132]. Modulation of adiponectin actions through the expression of adiponectin receptors may thus be a novel and promising therapeutic option.

### 7.5. Ghrelin

Ghrelin is a 28-amino-acid growth-hormone-releasing peptide secreted by the stomach [135]. It causes increased food intake and weight gain in rodents [136, 137]. But in obese subjects circulating levels are decreased. This observation is against a central role of ghrelin in the determination of common obesity [138, 139]. It is noteworthy that ghrelin infusion decreases BP by 5–10 mmHg, although it also increases SNS activity, perhaps through compensatory baroreflex activation [140]. The long-term central nervous system mediated cardiovascular actions of ghrelin are still unknown. A recent work of Freeman et al. demonstrated that chronic central ghrelin infusion reduces BP and HR despite increasing appetite and promoting weight gain in normotensive and hypertensive rats [141].

Ghrelin may also improve endothelial function mimicking phosphoinositol 3-kinase-dependent actions of insulin to stimulate production of NO by endothelial cells and restoring the endothelin 1/nitric oxide balance in patients with obesity-related MetS, as observed by Tesauro and coworkers [142–144].

### 7.6. Further Perspectives

The prevalence of the MetS is escalating worldwide. Sympathetic overdrive may be the common thread of visceral adiposity, HT, dyslipidemia, and glucose intolerance in the clinical diagnosis of the MetS [29]. MetS is thus characterized by sympathetic overdrive with outflow activation to both kidneys and adipose tissue among others. Although the mechanisms responsible for the initial activation of SNS are still to be fully elucidated, hyperinsulinemia, derangement of circulating adipokines, and beta receptor polimorphisms are all implicated and may cause the development of HT, I.R., diastolic dysfunction, and finally renal disease. Although lifestyle correction and hypotensive medications are the first-line therapy for obesity and HT in the MetS, interventions that target the SNS directly may be of further benefit. Such benefits may even be weight-unrelated and associated with a significant reduction in end-organ damage [37]. Afferent adrenergic signaling from the kidneys was recently identified as an important contributor to central SNS overdrive and SNS outflow to the kidneys is involved in cardiovascular, renal, and metabolic control [145, 146]. In recent clinical investigations, functional renal denervation obtained by means of catheter-based radiofrequency or ultrasound technologies achieved BP control in patients with resistant HT [147, 148] and polycystic ovary syndrome [149], further emphasizing the link between SNS overdrive and I.R., HT, and other comorbidities in the MetS. Finally, catheter-based renal denervation was investigated in end-stage renal disease (ESRD) patients with resistant intradialytic HT, who are supposed to compel a significant SNS overdrive [150, 151]. In preliminary investigations, a reduction of SNS overdrive with good control of blood pressure was obtained in small series of ESRD disease patients on maintenance hemodialysis by Di Daniele and coworkers [152], Ott et al. [153], and Schlaich and Coworkers [154].

Further investigations addressing the still open questions in the treatment of resistant HT and evaluating potential new indications such as the MetS or heart failure are still necessary to prove the safety and effectiveness of renal denervation in these patients. By modulating sympathetic activity, renal denervation may have the potential to provide significant benefits in a variety of diseases.

## Figures and Tables

**Table 1:** Effects of insulin resistance, ghrelin, and some adipokines on endocrine and metabolic functions in the pathogenesis of the MetS.

	General effects	Effects on sympathetic nervous system	*In vitro*/animal studies (references)	Human studies (references)
Insulin resistance	Direct antinatriuretic action	(i) Its intracerebral administration increases sympathetic outflow (ii) Induces sympathetic overactivity (iii) Stimulates SNS to increase cardiac output	[46–50]	[39, 49, 56–58]

Leptin	(i) Levels correlate with adipose tissue mass (ii) Satiating factor decreases food intake (iii) Physiological regulation of feeding behavior trough hypothalamic receptors	Vasocontractile effect related to SNS activation	[90, 92, 95, 97, 103, 104, 109, 110]	[90, 91, 99, 100, 102, 107, 111]

NEFAs	Levels are increased in obesity and inversely correlated with insulin sensitivity	Induce a central activation of MNSA in lean subjects	[115–118]	[114, 119, 122]

Adiponectin	(i) Levels are inversely related to obesity, DM, and insulin resistant states (ii) Ameliorates obesity-related hypertension		[127, 128, 132, 133]	[129, 131]

Ghrelin	(i) Its infusion decreases blood pressure and HR (ii) Improves endothelial function (iii) Promotes weight gain and increases appetite	Its infusion increases SNS activity	[136, 137, 141, 143]	[140, 142, 144]

## Abbreviations

MetS: Metabolic syndrome
CVD: Cardiovascular disease
BP: Blood pressure
HT: Hypertension
CKD: Chronic kidney disease
SNS: Sympathetic nervous system
RAAS: Renin-angiotensin system
I.R.: Insulin resistance
SNA: Sympathetic nerve activity
MSNA: Muscle sympathetic nerve activity
NE: Norepinephrine
AN: Arcuate nucleus
DM: Diabetes
HR: Heart rate
NO: Nitric oxide
PI3K: Phosphatidylinositol-3′-kinase
MAPK: Mitogenic-activated protein-kinase
NEFAs: Nonesterified fatty acids
STZ: Streptozotocin
HMW: High molecular weight
ESRD: End-stage renal disease
ROS: Reactive oxygen species.
